# Genetic and Epigenetic Adaptation Mechanisms of Sheep Under Multi-Environmental Stress Environment

**DOI:** 10.3390/ijms26073261

**Published:** 2025-04-01

**Authors:** Li Zhu, Lin Tang, Kang Zhang, Hongyu Nie, Xiao Gou, Xiaoyan Kong, Weidong Deng

**Affiliations:** 1 Yunnan Provincial Key Laboratory of Animal Nutrition and Feed Science, Faculty of Animal Science and Technology, Yunnan Agricultural University, Kunming 650201, China; zhuli18328815855@163.com (L.Z.); zero--xc@163.com (L.T.); 2 School of Animal Science and Technology, Foshan University, Foshan 528231, China; zhangkang39@outlook.com (K.Z.); 19984432910@163.com (H.N.); gouxiaosa@163.com (X.G.)

**Keywords:** sheep, environmental adaptation, genetic mechanisms, omics, high-altitude adaptation, cold adaptation, drought adaptation

## Abstract

Sheep (*Ovis aries*), domesticated from wild Asian mouflon ~10,000 years ago, are an important livestock species adapted to various ecological environments. Recent advancements in high-throughput sequencing and global environmental databases have facilitated the exploration of genetic–environmental associations, uncovering the genetic and epigenetic mechanisms behind sheep’s adaptation to multiple environments. Studies show that HIF-1α and *EPAS1* enhance high-altitude adaptation via hypoxic stress regulation; *UCP1* contributes to cold adaptation through non-shivering thermogenesis; *SLC4A4* and *GPX3* increase drought resistance by regulating renal water reabsorption; and *SOCS2* likely plays a role in metabolic and stress response regulation. Additionally, sheep adapt to temperature, drought, and environmental stress through DNA methylation, transcriptional regulation (e.g., *SOD1*, *GPX4*), heat shock proteins (e.g., *HSP70*), and metabolic pathways (e.g., *UCP1*). These findings offer valuable insights for improving sheep breeding and genetic enhancement. This review summarizes the mechanisms of adaptation to high altitude, cold, heat, drought, and comprehensive climate stress.

## 1. Introduction

Sheep (*Ovis aries*) were among the earliest domesticated livestock, with archaeological and genomic evidence tracing their domestication to ~10,000 years ago in the Fertile Crescent [1]. The Asiatic mouflon (*Ovis orientalis*) is widely recognized as their primary ancestor, though potential gene flow from the urial (*Ovis vignei*) remains uncertain [2]. By ~8000–9000 years ago, sheep had spread into Mesopotamia, Europe, and Asia, with key genetic exchanges during the Bronze and Iron Ages (~5000–3000 years ago), accelerating their global spread and adaptation [3]. Initially domesticated for meat, sheep later became a key source of wool and dairy, driving specialized breeding [4]. Beyond their economic role, they shaped early trade, cultural, and agricultural systems [5]. Today, they inhabit diverse ecosystems, exhibiting extensive genetic and phenotypic diversity shaped by natural selection and artificial breeding.

Advances in multi-omics technologies have enhanced our understanding of sheep adaptation. Genomic studies reveal key adaptations to high altitude, cold, and arid environments, with *EGLN1* and *HIF-1α* aiding hypoxia tolerance, *UCP1* in thermoregulation, and *GPX3* in water metabolism [6,7]. Population genetics identifies strong selection signals and historical gene flow in different sheep populations [8]. Transcriptomics clarifies gene expression under environmental stress, while epigenomics demonstrates the role of DNA methylation and histone modifications in adaptation [9,10]. The integration of these datasets has provided new insights into the complex networks driving adaptation across diverse environments [11,12,13].

This review examines the genetic and epigenetic mechanisms underlying sheep adaptation to diverse environmental stressors. By integrating genomics, transcriptomics, epigenomics, and metabolomics, we highlight key adaptive pathways, including HIF signaling for hypoxia tolerance, thermogenic regulation for cold adaptation, and water metabolism genes in arid environments. Furthermore, we identify both conserved and lineage-specific genetic signatures through population genomics and selective sweeps. By synthesizing multi-omics data, this review not only identifies existing knowledge gaps but also underscores the potential of integrative omics and genome-assisted selection in developing climate-resilient sheep breeds, ultimately contributing to sustainable livestock production in a changing global climate.

## 2. Diversity, Distribution, and Adaptation of Sheep Breeds

**Figure 1:**
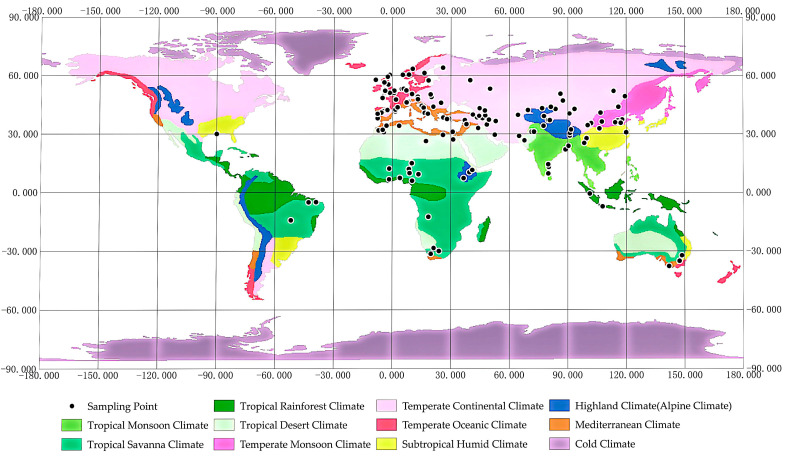
Climate distribution of resequencing samples of major sheep breeds.

Sheep are one of the most widely distributed livestock species and are adapted to extreme environments such as high altitude, cold, heat, and drought. These complex ecological gradients have created diverse survival pressures, driving sheep to exhibit remarkable genetic and physiological adaptations through long-term natural selection [14,15]. These pressures not only shape survival strategies but also provide valuable resources for studying biological adaptation mechanisms (Figure 1) [16].

In high-altitude regions like the Tibetan Plateau (elevation 3500–5000 m), Tibetan sheep have adapted to low oxygen, UV radiation, and cold through the regulation of the HIF pathway and enhanced antioxidant capacity [6,7,17]. Similarly, Ethiopian Menz sheep exhibit selection in genes like *PPP1R12A* and *RELN*, which are associated with respiratory adaptation, indicating convergent evolution in high-altitude resilience [15]. In cold regions like the Mongolian Plateau and Northern China, Mongolian and Tan sheep enhance cold tolerance through fat storage and non-shivering thermogenesis, while Small-Tailed Han sheep exhibit both reproductive resilience and cold resistance in extreme climates [18,19,20]. Beyond Asia, Yakut, Baikal, Tuva, and Changthangi sheep exhibit thermogenic and metabolic adaptations, reflecting the widespread evolution of cold tolerance in sheep [21,22,23]. In hot regions like southern China and Africa, Hu sheep regulate water metabolism for thermoregulation, while fat-tailed sheep rely on tail fat reserves for energy storage and heat tolerance [24,25,26]. Globally, heat adaptation strategies vary, including heat shock protein regulation (Indian and Macheri sheep) [27,28], metabolic flexibility (Hu and Egyptian sheep) [29,30], spermatogenesis protection (Turpan black sheep) [31], and pigmentation-linked thermoregulation (Iranian sheep), highlighting genetic diversity in thermal stress resilience [32]. Drought adaptation in sheep involves diverse strategies across regions. Tan and Altay sheep enhance renal water reabsorption, while Egyptian fat-tailed sheep rely on fat metabolism for energy conservation. Taklimakan desert and Xinjiang sheep regulate osmotic balance and feed efficiency, highlighting global genetic adaptations to arid environments [33,34,35,36]. Certain breeds, such as fine-wool sheep, are both cold- and drought-tolerant, maintaining high-quality wool production across diverse ecological zones [37]. Expanding the discussion to globally distributed breeds provides a comprehensive perspective on genetic mechanisms driving environmental adaptability, contributing to sustainable breeding strategies for climate resilience.

## 3. Methods for Studying Environmental Adaptation

**Figure 2:**
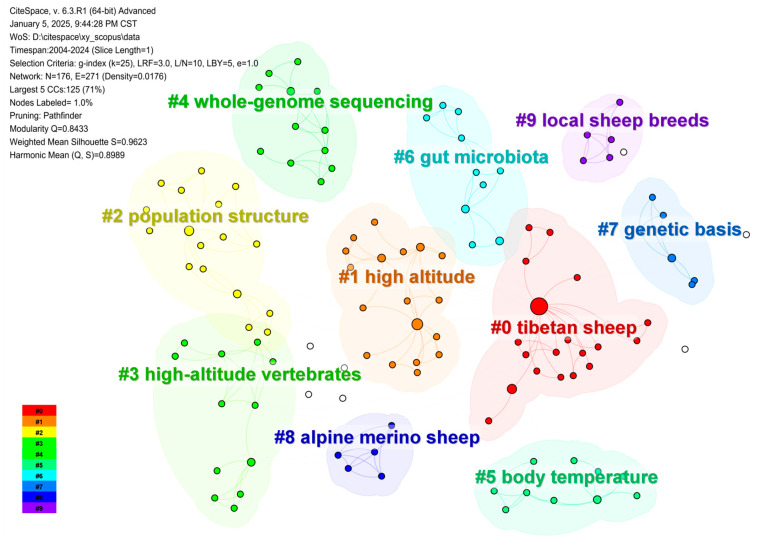
Keyword co-occurrence network analysis of environmental adaptation research (2004–2024).

We conducted bibliometric analysis using CiteSpace 6.3.R1, with data spanning from 2004 to 2024 [38]. A 1-year time slice was applied, focusing on “Keyword” nodes with g-index (k = 25) for collaboration networks [39]. The “Pathfinder” cutting method was used to visualize keyword co-occurrence, and the Log-Likelihood Ratio (LLR) algorithm was employed to cluster keywords [40]. This analysis identified key research themes and trends in environmental adaptation, highlighting the focus on genetic mechanisms, ecological adaptability, and climate resilience (Figure 2).

Whole genome sequencing (WGS) and pangenomics provide essential data for understanding environmental adaptation in sheep. Niu et al. (2024) identified 35 adaptation-related genes, including *HOXA10* and *JAZF1* (fat tail formation), *FER* and *FGF5* (wool traits), and *RXFP2* (horn morphology), by performing whole-genome sequencing on 266 sheep across 18 regions [41]. Missense mutations in *RXFP2* and *PAPSS2* were strongly linked to high-altitude adaptation, influencing skeletal morphology and metabolic processes [41]. Pangenomics provides critical insights into the interplay of core and variable genomes, where core genes ensure stability and variable genes contribute to ecological stress resistance, supporting sheep adaptation to diverse environments [12,42]. Population genetic studies using tools like ADMIXTURE and STRUCTURE have uncovered stratification among sheep populations [33,43,44]. Strong selection signals were found in *EPAS1* and *EGLN1* for highland sheep, particularly in Tibetan and Mongolian populations [17,45,46]. Gene flow between Xinjiang fine-wool sheep and other populations facilitated the dissemination of adaptive traits [43,45]. Contributions from wild species also enriched traits like metabolism, cold tolerance, and disease resistance. Genome-Wide Association Study (GWAS) has been pivotal in identifying the genetic bases of adaptive traits [34,43]. Studies have identified genes like *ADIPOQ* and *TSHR* linked to lipid metabolism and thermogenesis, contributing to sheep adaptation in extreme climates [47,48]. Additionally, domestication-related genes such as *GDF9* and *BMP15* were associated with enhanced reproductive capacity [49]. Landscape genomics integrates environmental variables with genomic data to link genes with ecological conditions [45,50,51]. Genes like *EGLN1* (high-altitude) [46] and *UCP1* (cold regions) show strong associations with environmental factors. Recent advancements in machine learning have facilitated the integration of environmental variables, such as temperature and precipitation, with genomic data to study adaptive traits in sheep.

Multi-omics approaches provide critical insights into sheep adaptation to environmental stresses, including temperature [32], aridity [35], UV radiation [52], and hypoxia [10]. Genomics and selection signal analyses have identified key genes (*MC1R*, *HMOX2*, *BMP2* and *PDGFD*) linked to pigmentation [53], oxygen transport [7] and energy storage and insulation [54]. Epigenetic studies (e.g., ATAC-Seq and ChIP-Seq) have revealed the role of enhancers and promoters in regulating water retention and oxygen sensing, with the *VEGFA* gene implicated in adipose tissue homeostasis, which is crucial for maintaining energy balance and insulation [11]. Transcriptomic and metabolomic studies reveal that Tibetan sheep adapt to high-altitude environments by reducing lipid metabolism, enhancing cardiac function, regulating fluid balance, and boosting immunity and antioxidant capacity [55]. Microbiome studies found that Bacteroides and Prevotella enhance fiber digestion and energy production, aiding survival in arid conditions [56]. These findings highlight multi-omics as a vital tool for understanding and improving sheep’s environmental resilience.

Advanced computational tools, such as AlphaFold [57] for protein structure prediction and CRISPR-Cas9 [58] for gene validation, have revolutionized adaptation research in sheep. Integration of genomic, transcriptomic, and environmental data enables precise phenotypic predictions [54], while WGS and pangenomics uncover key adaptive mechanisms, including *EGLN1* for high-altitude adaptation [17] and *UCP1* for cold tolerance [18]. These advancements, combined with machine learning and multi-omics approaches, provide powerful strategies to enhance livestock resilience and sustainability under climate challenges. To further illustrate the key methodologies applied in sheep adaptation research, we summarize major research approaches and their applications in Table 1.

## 4. Mechanisms of Adaptation in Sheep

### 4.1. Mechanisms of Hypoxia Adaptation

**Figure 3:**
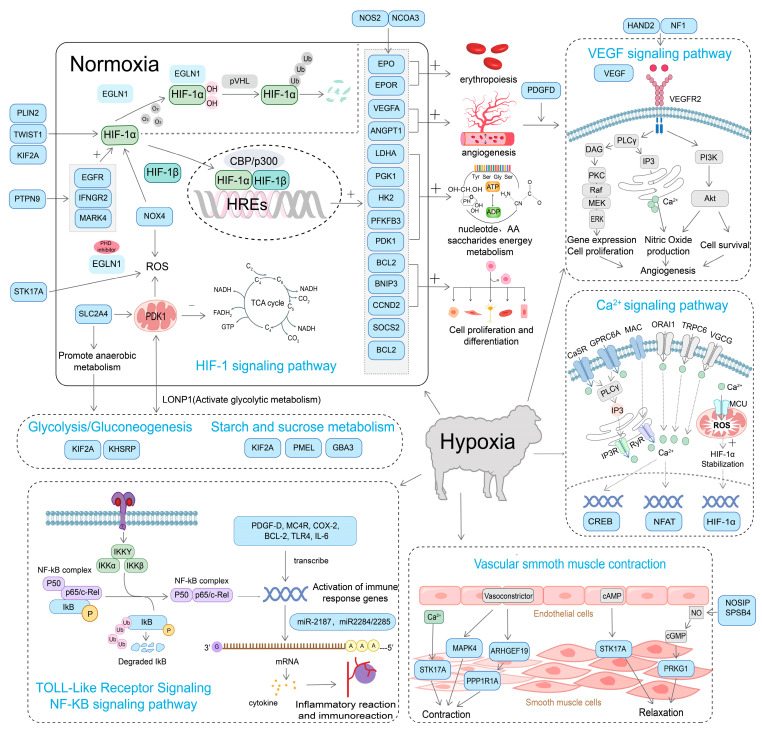
Mechanisms of hypoxia adaptation in sheep.

Sheep have developed genetic and physiological adaptations to high-altitude hypoxia, providing a valuable model for investigating mechanisms of oxygen homeostasis and hypoxia tolerance [10,83] (Table 2). Advances in genomics, transcriptomics, proteomics, and epigenetics have provided multi-level insights into these adaptive mechanisms (Figure 3). Hypoxia-inducible factors (HIFs), such as *EPAS1* (HIF-2α) and HIF-1α, regulate key genes like *EPO*, *VEGF*, and *PDK1*, which enhance oxygen transport, angiogenesis, and energy metabolism [84]. Tibetan sheep, for instance, show strong selection signals in the *EGLN1* gene, which stabilizes *HIF-1α* and *HIF-2α*, while genes like *ANGPTL4* and *ADAM17* contribute to vascular growth and oxygen supply [17]. Beyond genetic factors, epigenetic modifications significantly influence hypoxia adaptation. EPAS1 methylation modulates oxygen metabolism, optimizing gene expression for hypoxic conditions [85]. Additionally, histone modifications and non-coding RNAs (miRNAs, lncRNAs) regulate angiogenesis, energy metabolism, and erythropoiesis, enhancing high-altitude resilience [11,71].

Comparative studies with yaks (*Bos grunniens*) reveal convergent evolution in hypoxia-related pathways [86]. Both Tibetan sheep and yaks show selection in *EPAS1, EGLN1*, and PRKAA1, key regulators of the AMPK pathway for hypoxic energy metabolism [87]. However, yaks show additional enhancements in mitochondrial oxidative phosphorylation efficiency, suggesting species-specific adaptations in hypoxia tolerance [88]. Such comparisons provide an evolutionary perspective on high-altitude resilience and highlight adaptive introgression as a potential mechanism driving hypoxia adaptation in domestic sheep [89].

In response to hypoxia, genes such as *SOD2* and *GPX1* help reduce oxidative stress, while hemoglobin-related genes (*HBA*, *HBB*) increase oxygen-carrying capacity [17,83]. Post-translational modifications regulate hypoxia adaptation by modulating oxidative stress and oxygen transport, as seen in Tibetan sheep proteomic analysis (*HBB*, *PRDX2*, *GPX1*) [84]. Proteomic studies have identified critical proteins in oxygen transport, vascular development, and energy metabolism, such as *HBB*, *PRDX2*, *GPX1*, *VEGFA,* and *LTBP4* [84]. Gut microbiota enhances hypoxia adaptation in Tibetan sheep by increasing Prevotellaceae-mediated volatile fatty acid production for energy metabolism [90,91,92].

Integrative multi-omics analyses have deepened our understanding of hypoxia adaptation [7]. Genetic selection in *EPAS1*, *EGLN1*, and *PRKAA1* underpins genetic adaptation, while post-translational modifications and microbiota shifts enhance physiological resilience [93,94]. Future CRISPR/Cas9 and high-resolution omics studies will further refine these mechanisms for high-altitude livestock improvement.

**Table 2:** Overview of known genes under local adaptation for hypoxia in sheep populations.

Pouplation	Genes	Function	References
**Tibetan sheep**	*EPAS1*, *EGLN1*, *HIF1A*, *VEGFA*, *EPO, HBB*	*EPAS1*, *EGLN1*, and *HIF1A* regulate the HIF pathway; *VEGFA*, *HBB*, *HBA*, and *EPO* enhance oxygen transport	[89,95,96]
**Andean sheep**	*HMOX1*, *NOS3*, *VEGFA*	*HMOX1* and *NOS3* modulate CO and NO signaling to regulate pulmonary vascular tone; *VEGFA* promotes vascular remodeling	[97,98]
**Ethiopian sheep**	*PPP1R12A*, *RELN*, *PARP2*, *DNAH9*, *SDK1*, *ARMC3*, *PRDM16*, *COL6A3*, *COL25A1*	*PPP1R12A*, *RELN*, *PARP2*, **and** *DNAH9* **regulate respiratory system development, oxygen transport, and cellular responses to hypoxia; *SDK1, ARMC3, PRDM16, COL6A3*, and *COL25A1* regulate oxygen transport, thermogenesis, and vascular remodeling to enhance hypoxia adaptation**	[15,99]
**Mongolian sheep**	*DYSF*, *EPAS1*, *JAZF1*, *PDGFD*, *NF1*	Enhance hypoxia response, vascular function, and energy metabolism for high-altitude adaptation	[33]

### 4.2. Molecular Adaptations to Ultraviolet Radiation

The adaptation of sheep to UV radiation is achieved through multi-level genetic regulatory and epigenetic regulatory mechanisms. In Changthangi sheep, key genes such as *TYR*, *TYRP1*, and *DCT* enhance melanogenesis, effectively increasing the skin’s protection against UV radiation [100]. Epigenetic modifications such as DNA methylation in MC1R and TYR influence melanin production, regulating pigmentation patterns in high-altitude sheep [101]. Additionally, histone modifications in SLC45A2 have been linked to melanocyte differentiation, further enhancing UV protection [102]. Tibetan sheep regulate pigment deposition through *MC1R* and *MITF*, while *LEF1* and *GPX1* genes cooperate to enhance antioxidant capacity and repair UV-induced damage [103,104]. Studies suggest that long non-coding RNAs (lncRNAs) modulate UV response genes, influencing skin pigmentation and oxidative stress resilience [105].

At the proteomic level, the *SLC45A2* gene plays a crucial role in melanogenesis, further enhancing the sheep’s tolerance to UV radiation [106,107]. Ouled Jellal sheep exhibit epigenetic regulation of *SDF4*, which promotes cell proliferation and survival, effectively mitigating UV-induced cellular damage [108]. In Egyptian fat-tail sheep, *TGM3*, *RAD54L*, *CHEK2*, and *MUTYH* support epidermal integrity, DNA repair, and oxidative stress defense [36]. These findings highlight the coordinated regulation of genes and proteins in UV adaptation across sheep breeds. Across different sheep breeds, multiple genes contribute to UV adaptation through pigmentation, antioxidant mechanisms, and DNA repair, as summarized in Table 3.

### 4.3. Adaptation Mechanisms to Temperature Variations

#### 4.3.1. Cold Adaptation Mechanisms

**Figure 4:**
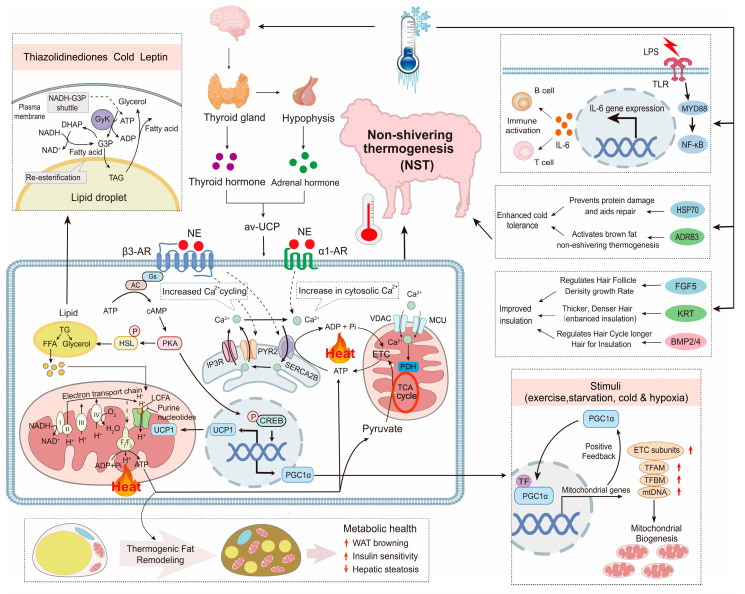
Molecular mechanisms of cold adaptation in sheep. Red arrows indicate increased expression or activity levels of the corresponding components.

Sheep adapt to cold environments through molecular and metabolic regulation, including both UCP1-dependent and independent thermogenesis (Figure 4).

*UCP1* in brown adipose tissue converts energy into heat to maintain body temperature, with Altay sheep primarily relying on UCP1-dependent pathways, while Hu sheep use non-UCP1 mechanisms regulated by *SERCA* and *CKM* [18]. Genes like *BMPR1B* and *PRDM16* promote adipose browning, enhancing cold resistance [94]. Transcriptome analysis of Altay and Hu sheep under cold exposure identified PPAR (*APOC3*, *LPL*, *FABP4*) and cAMP (*ADCY10*, *ADORA2a*) pathways as key regulators of fatty acid metabolism and thermogenesis [110]. Additionally, BMP2 and BMP4 contribute to adipogenesis and thermogenic activation, facilitating cold adaptation in fat-storing tissues [111]. *ATP2A1* and *SLN* were involved in calcium signaling-based non-shivering thermogenesis, further supporting energy balance and heat production under cold stress [112,113]. FGF5 regulates hair follicle cycles by promoting the transition from growth to regression, with loss-of-function mutations leading to longer wool fibers, enhancing insulation in cold-adapted sheep [114]. Cold adaptation in sheep is driven by a diverse set of genes regulating thermogenesis, lipid metabolism, and energy homeostasis across different populations (Table 4).

Cold exposure also triggers oxidative stress and immune modulation. Cold stress reduces serum immunoglobulin levels and increases pro-inflammatory cytokines (e.g., *IL-6*, *TNF-α*), reflecting suppressed immune function [115]. Cold stress upregulates HSP70 family genes (*HSPA6*, *HSPA8*), providing tissue protection and modulating inflammation [116]. Rumen microbiota, particularly Lactobacillus and Prevotella, enhance fiber digestion and SCFA production, supporting energy needs during cold seasons [117]. Host genes (*FASN*, *CPT1A*) regulate lipid metabolism and fatty acid oxidation, ensuring energy efficiency and antioxidant defense under cold stress [118]. *ADRB3* also plays a role in lipid mobilization, promoting fat breakdown and thermogenic activation, further enhancing cold resistance in sheep [18].

**Table 4:** Overview of known genes under local adaptation for cold in sheep populations.

Pouplation	Genes	Function	References
**Tibetan sheep**	*FKBP5*, *PLSCR4*, *CDH8*, *HSPA1A*, *HSPB1*, *HSPD1*, *HSF4*	*FKBP5*, *PLSCR4*, and *CDH8* contribute to thermogenesis; *HSPA1A*, *HSPB1*, and *HSPD1* enhance cold and hypoxia tolerance	[7,103]
**Mongolian sheep**	*LEP*, *UCP1*, *PGC-1α*, *CIDEA*, *COX4*, *PM20D1*	*LEP* regulates metabolism; *UCP1* drives WAT browning; *PGC-1α* enhances mitochondrial biogenesis; *CIDEA* and *COX4* mark WAT browning; *PM20D1* contributes to alternative thermogenic pathways	[20]
**Yakut sheep**	*UCP1*, *HSP90AA1*, *FOXO1*	*UCP1* **and** *HSP90AA1* **support thermogenesis and cold protection;** *FOXO1* **regulates energy metabolism and antioxidant responses**	[119]
**Baikal sheep**	*DDB2*, *SOCS6*	*DDB2* supports DNA repair, while *SOCS6* regulates metabolism for cold adaptation	[22,23]
**Tuva sheep**	*GLIS1*, *AADACL3*, *GPR179*	*GLIS1* regulates cell differentiation, *AADACL3* promotes fat deposition for energy storage, and *GPR179* contributes to visual adaptation in cold environments	[22,23]
**Changthangi sheep**	*UCP2*, *UCP3*	*UCP2* and *UCP3* enhance thermogenesis, lipid metabolism, and oxidative stress resistance	[21]
**Altay sheep**	*UCP1*, *ADRB3*, *ADORA2A*, *ATP2A1*, *RYR1*, *IP6K1*	*UCP1*, *ADRB3*, and *ADORA2A* drive thermogenesis and lipid metabolism, while *ATP2A1*, *RYR1*, and *IP6K1* regulate calcium signaling and energy balance, ensuring cold adaptation.	[18]

#### 4.3.2. Heat Adaptation Mechanisms

Heat stress triggers cellular stress responses and thermoregulation mechanisms, enabling sheep to maintain survival and productivity in high-temperature environments. Heat shock proteins (HSPs) are central to heat stress responses. *HSP70* and *HSP90* enhance cellular tolerance to heat stress by stabilizing proteins and inhibiting apoptosis [120,121]. Moreover, the *HIF1α* gene plays a key role in regulating oxygen metabolism and energy balance during heat stress in sheep, while genes such as *PRLR* and *TNFAIP3* are involved in cellular adaptation via signaling pathways [122,123]. To mitigate heat stress, sheep upregulate glycolysis and lipid metabolism-related genes (e.g., *PPARG*, *ACADM*), providing energy to support cell survival [120]. Enhanced short-chain fatty acid (SCFA) metabolism in the rumen improves metabolic flexibility and supports adaptation to high temperatures [124]. Heat stress disrupts immune function, increasing pro-inflammatory cytokines (e.g., *IL-6*, *IL-10*) and reducing anti-inflammatory factors (e.g., *TGF-β*), exacerbating inflammatory responses [125]. Reactive oxygen species (ROS) accumulation induces oxidative stress, which sheep counteract by upregulating antioxidant enzymes like *SOD* and *CAT* [126]. Behavioral adaptations, such as reduced activity, seeking shade, and increased evaporative cooling, help sheep regulate body temperature under heat stress [127,128]. Heat stress impacts rumen fermentation and nutrient utilization in sheep, leading to changes in volatile fatty acid (VFA) production, which support energy metabolism and adaptation to high-temperature conditions [129,130]. The genetic basis of heat adaptation in sheep involves pathways related to thermotolerance, metabolism, and oxidative stress regulation, with population-specific variations in key adaptive genes (Table 5).

#### 4.3.3. Drought Adaptation Mechanisms

Studies have shown that indigenous sheep populations in Xinjiang regulate the expression of *GPX3* and *GPX7* to enhance antioxidant capacity, thereby reducing water loss. Additionally, *SLC4A4* and *ECE1* mediate water–salt balance, optimizing renal water reabsorption to facilitate adaptation to extreme drought conditions [34]. Furthermore, Egyptian fat-tailed sheep have evolved unique metabolic adaptations, where *PCK1* and *ACAA2* enhance gluconeogenesis and fatty acid metabolism, improving energy efficiency while minimizing water consumption. Meanwhile, *HSP70* and *HSP90* function as heat shock proteins that stabilize proteins under thermal stress, ensuring cellular survival [36]. Whole-genome resequencing analyses further highlight the roles of *BANK1* and *TSHR* in regulating energy metabolism and heat tolerance, contributing to the survival of indigenous sheep in arid environments [35]. Collectively, these genetic adaptations have played a crucial role in shaping the resilience of sheep to extreme arid conditions, providing valuable insights for breeding programs aimed at improving drought-resistant sheep populations [33]. Sheep exhibit remarkable adaptability to arid environments through a combination of genetic, physiological, and behavioral mechanisms (Table 6).

### 4.4. Integrated Environmental Adaptation Mechanisms

Sheep have developed diverse adaptations to hypoxia, cold, UV radiation, heat, and drought, enabling survival in high-altitude and arid environments. In high-altitude regions, genes such as *EPAS1*, *EGLN1*, and *HIF1A* enhance oxygen transport, while HSPs aid cold tolerance and *MC1R*, *TYR* regulate pigmentation for UV protection [34,131]. In deserts, HSP70 and HSP90 stabilize proteins under heat stress, *AQP* genes manage water retention, and *FOXO1* supports fat metabolism for drought adaptation [3].

To identify genetic signatures of these adaptations, environmental–genomic approaches such as PCA and ENMs integrate climate variables with genomic data, linking key environmental factors to adaptive traits [51]. For instance, Gheyas et al. identified genomic regions associated with temperature and water scarcity adaptation. Recognizing conserved adaptive pathways, such as thyroid hormone regulation, provides insights into multi-trait adaptation [132]. The integration of multi-omics and ecological modeling facilitates the discovery of adaptive variants, supporting climate-resilient breeding strategies for improved sheep productivity under environmental stress.

## 5. Main Findings and Discussion

### 5.1. Identification and Functional Analysis of Adaptive Genes

Sheep exhibit diverse genetic adaptations to extreme environments, driven by natural selection and the genetic variability within key adaptive genes. Our analysis highlights how genetic variability in critical genes across various environmental stressors, including hypoxia, cold, drought, heat, and UV radiation, enables sheep to adapt to different environments. These genetic differences are essential for enabling populations to thrive in specific ecological niches, as they influence the functional outcomes of adaptation. In high-altitude hypoxic conditions, *EPAS1*, *HIF1A*, and *EGLN1* regulate the HIF pathway, which is responsible for enhancing oxygen transport and metabolic adaptation [89,95,96]. The genetic variability within these genes allows sheep populations to have different responses to varying levels of oxygen, which is particularly critical in high-altitude environments. For instance, *EPAS1* has undergone convergent evolution across multiple high-altitude domestic species, including Tibetan sheep, yaks, Tibetan cattle, and Tibetan pigs, showing that genetic diversity within this gene contributes to the adaptability of different species to low-oxygen environments. This genetic variability provides the flexibility to modulate hypoxia-related pathways in different ways, enhancing survival and reproductive success at different altitudes [86]. In cold environments, *UCP1*, *UCP2*, and *UCP3* facilitate thermogenesis [20,119], and the genetic variability in these genes influences the effectiveness of non-shivering thermogenesis across sheep breeds. Similarly, *LEP* and *PGC-1α* [20] support lipid metabolism, with their genetic variations determining the extent of cold tolerance in various populations. Similar mechanisms are observed in reindeer (*Rangifer tarandus*) and muskox (*Ovibos moschatus*), which also rely on UCP1-mediated non-shivering thermogenesis to withstand extreme cold [133]. However, Tibetan sheep exhibit unique lipid metabolism adaptations, resembling those of yaks rather than other sheep breeds [134]. In arid environments, *GPX3*, *GPX7*, *ANXA6*, *PTGS2*, *CPB1*, and *CPVL* regulate water–salt metabolism and oxidative stress resistance, ensuring efficient water usage [34]. Comparable adaptations occur in dromedary camels, where AQP genes enhance water retention, while Kazakh sheep and Bactrian camels share selection signals in SLC4A4, aiding in sodium balance under drought conditions. Additionally, in hot environments, *HSP70* and *HSP90* mitigate oxidative damage and support heat stress resistance [28]. These genes are also critical for cattle, where strong selection in HSP90AA1 enables thermotolerance [3]. Notably, Egyptian fat-tailed sheep exhibit higher HSP expression, similar to that of desert-adapted goats, enhancing cellular stress tolerance [135]. For UV adaptation, genes such as *MC1R*, *MITF*, and *GPX1* help protect against UV damage and maintain skin integrity [104,109]. Comparable pigmentation adaptations are seen in horses and cattle, where ASIP and MC1R variants contribute to coat color variation under high UV exposure [136]. Tibetan sheep exhibit strong ASIP selection, resembling the dark pigmentation patterns of Tibetan cattle and goats [137]. Reproductive adaptation is vital for survival at high altitudes, where *PAPPA* and *BMPR1B* regulate follicle growth and litter size. The genetic variability in these genes allows Tibetan sheep to optimize reproductive success under hypoxic conditions. Specifically, *PAPPA* influences dominant follicle development, and *BMPR1B* undergoes splicing and genetic variations that help improve reproductive success in response to environmental challenges [94]. Multi-omics analyses further reveal cell-type-specific expression and epigenetic modifications, highlighting key mechanisms of reproductive adaptability under hypoxia conditions [3]. These adaptive mechanisms, driven by genetic variability within key genes and their regulatory pathways, are critical for sheep survival across diverse environments. The presence of genetic diversity within these genes offers valuable targets for molecular breeding programs aimed at improving climate resilience and enhancing productivity across different ecological conditions.

### 5.2. Integration of Signaling Pathways

Cooperated signaling pathways play a crucial role in sheep adaptation to diverse environmental stressors. The HIF signaling pathway is central to high-altitude adaptation, with *EPAS1*, *EGLN1*, and *HIF1A* enhancing oxygen transport and mitochondrial efficiency [87,138], similar to yaks [88]. Additionally, the VEGF and PPAR signaling pathway further promotes survival at high altitudes [6]. To combat cold and UV-induced oxidative stress, the NRF2 antioxidant pathway mitigates ROS damage, while Ca^2^⁺ signaling regulates heat shock protein (HSP) responses, mitochondrial function, and energy production, supporting temperature stress resilience [139]. In cold climates, UCP1-dependent thermogenesis, modulated by calcium and cAMP pathways, facilitates heat production and metabolic adaptation, a mechanism also observed in reindeer and yak [18]. In hot and arid environments, PPAR signaling enhances lipid oxidation and water conservation, optimizing metabolic efficiency for drought resilience [140,141,142]. AMPK signaling maintains energy homeostasis, a crucial mechanism for both high-altitude and arid-adapted breeds [143]. Immune resilience is essential for sheep to survive in extreme climates. JAK/STAT and NF-κB pathways regulate adaptive immune responses, with selection signals in Tibetan and Andean sheep suggesting immunogenetic modifications to counteract hypoxia-induced immunosuppression [144,145]. Meanwhile, mTOR signaling governs autophagy and cell proliferation under nutrient limitations, a key factor in resource-scarce environments such as high-altitude grasslands and semi-arid regions [68,146]. These integrated signaling networks collectively optimize stress response, metabolic efficiency, and physiological adaptation, ensuring sheep survival and productivity across extreme environments. Future research should explore single-cell transcriptomics and CRISPR-based functional validation to elucidate species-specific signaling adaptations and enhance livestock resilience through genetic selection.

### 5.3. Epigenetic and Microbiome Regulation

Epigenetic modifications and gut microbiota composition are key to sheep adaptation. High-altitude hypoxia or drought induces DNA methylation and histone modifications, regulating stress–response genes. Demethylation of HIF1A and EPAS1 enhances oxygen transport and metabolism [13]. Methylation of PPARGC1A and GDF9 influences follicular development and ovulation, adjusting reproductive strategies to environmental stress [147]. Similarly, histone acetylation in BMP15 and FSHR modulates hormone signaling, promoting prolificacy in high-fecundity breeds [148]. The gut microbiome adapts to optimize nutrient absorption and metabolism. At high altitudes, Lactobacillus and Bacteroides enhance digestion and SCFA production [64], improving energy efficiency under hypoxia. SCFAs also act as epigenetic modulators, regulating metabolism and immune responses [67]. In arid conditions, microbial shifts maintain intestinal integrity and aid water conservation. The combined regulation of gene expression through epigenetics and microbiome interactions, such as through PPARG and FASN, optimizes energy efficiency and oxidative stress resistance, highlighting the integrated role of these systems in supporting sheep’s survival under harsh environmental conditions [62,63].

## 6. Conclusions and Future Perspectives

Sheep are highly adaptable to extreme environments, including high-altitude hypoxia, cold, and drought. Key genes such as *EPAS1* and *HIF1A* enhance hypoxia tolerance, while *UCP1* and *HSP70* facilitate cold adaptation through thermogenesis and stress responses. In drought conditions, genes like *GPX3* and *SLC4A4* improve water reabsorption. However, environmental adaptation in sheep involves complex, multi-level regulatory networks, not just single-gene effects. While genomics and epigenetics have provided insights, functional validation of candidate genes and environmental factors remains a challenge.

Future research should explore unique adaptive mechanisms in sheep that contribute to their survival in extreme environments. For instance, how high-altitude sheep develop metabolic strategies to combat hypoxia beyond the known HIF pathway, or how drought-resistant breeds optimize renal function for water conservation. Understanding these species-specific adaptations can provide deeper insight into evolutionary biology and improve breeding programs. Additionally, integrating genomics, transcriptomics, metabolomics, and epigenomics can help construct dynamic regulatory networks to uncover novel adaptation strategies. Gene-editing technologies like CRISPR/Cas9, AI, and protein structure prediction tools (e.g., AlphaFold) can further refine our understanding of these mechanisms. Time series studies and comparative genomics across species will help reveal shared adaptive strategies and their evolutionary significance.

In livestock production, genomic selection (GS) and marker-assisted selection (MAS) techniques can accelerate the breeding of sheep adapted to extreme environments, supporting sustainable agriculture and food security. Studies on indigenous sheep genetic variation also offer insights into conservation and productivity optimization. As climate change poses new challenges, these research efforts will not only improve livestock management but also contribute to the broader understanding of biological adaptation.

## Figures and Tables

**Table 1:** Research levels and methods in sheep studies.

Research Method	Combined Description	References
**Genome + Transcriptome**	Identified genes linked to morphological and agronomic traits	[8]
**Transcriptome + Metabolome**	Genetic and metabolic mechanisms of Tibetan sheep’s high-altitude adaptation	[59]
**Transcriptome + Proteomics**	DEPs and transcriptomic profiles reveal hair follicle development mechanisms	[60]
**GWAS + Transcriptomics**	Multi-tissue transcriptomes reveal the genetic basis of wool and growth traits	[61]
**Genome + Transcriptome + Population Genomics**	β-globin A boosts O_2_ affinity; *EGLN1* aids hypoxia adaptation	[13]
**Genome + Transcriptome + Land Genomics**	Selective sweeps reveal drought, hypoxia, and cold tolerance in Xinjiang	[35]
Epigenomics + Transcriptome	DNA methylation regulates ovarian gene expression and prolificacy in Hu sheep	[62]
Transcriptome + Epigenomics + GWAS	Gene regulation and methylation shape wool traits; epigenome reveals domestication	[11,63]
Single-cell Transcriptomics + Metagenomics	Rumen microbiome reveals the genetic basis of fermentation	[64]
Transcriptomics + Metabolomics	DEGs and pathways in sheep fat tails reveal *BMP2*’s role in adipogenesis and metabolism regulation	[65]
ATAC-seq + RNA-seq	Key pathways and genes regulating SMSC differentiation	[66]
WGS + RNA-Seq + ATAC-Seq + scRNA-Seq	Time-resolved multi-omics analysis reveals gene regulation in response to high-altitude hypoxia	[10]
Metagenomics + Metabolomics	3580 microorganisms, 732 metabolites identified; key metabolites: 4,6-isocanedione; adaptation in Hu sheep	[67]
**WGS**	Discovered adaptive mutations in *EPAS1*, *EGLN1* genes	[17,33,68]
**GWAS**	SNPs associated with body size traits in Hu sheep were identified and verified through luciferase reporter assays	[69]
**Lnc RNA-miRNA**	Highlighted hypoxia-induced lncRNA and miRNA roles	[70,71,72,73]
**mRNA**	Reveals prolificacy-related genes in high- and low-fecundity	[74]
**circRNAs-miRNAs-mRNA**	Regulates ovine hair follicle morphogenesis and adipogenic differentiation	[75,76]
**ChIP-Seq + Methylation profiling**	Unveiled epigenetic mechanisms in environmental adaptation	[77]
**LC-MS/MS proteomics**	Identified 245 proteins in ovine serum, used ZooMS marker for early domesticated sheep	[78,79]
**DEPs in tail adipose**	Regulate fat deposition via metabolic and PPAR pathways.	[80]
**GC-MS + LC-MS metabolomics**	372 metabolites identified in AGF; key pathways include amino acid and CoA metabolism	[81]
**metabolites**	107 metabolites; Hu sheep show better heat tolerance with key biomarkers	[82]

**Table 3:** Overview of known genes under local adaptation for UV in sheep populations.

Pouplation	Genes	Function	References
**Changthangi sheep**	*TYR*, *TYRP1*, *DCT*, *SLC45A2*, *PMEL*, *MLANA*	Regulate melanin biosynthesis and pigmentation, enhancing UV protection in high-altitude	[21,100]
**Tibetan sheep**	*MC1R*, *LEF1*, *MITF*, *GPX1*, *COL3A1*, and *CYPI7B1*	*MC1R* and *MITF* control pigmentation; *LEF1*, *GPX1*, and *CYPI7B1* enhance UV protection; *COL3A1* maintains skin elasticity and repairs UV damage	[17,68,104,109]
**Ouled Jellal sheep**	*SDF4*	*SDF4* protects against UV damage and supports cell proliferation	[108]
**Egyptian fat-tail sheep**	*RCC3*, *TGM3*, *RAD54L*, *CHEK2*, *MUTYH*, *CMPK1*, *TP53INP1* and *PRDX1*	UV adaptation, facilitating skin barrier formation, DNA repair, and oxidative stress defense under prolonged UV exposure	[36]

**Table 5:** Overview of known genes under local adaptation for heat in sheep populations.

Pouplation	Genes	Function	References
**Indian sheep**	*HSP70*, *HSP90*	Enhance thermotolerance by stabilizing proteins and reducing heat stress	[28]
**Turpan black sheep**	*SYCP2*, *TDRD9*, *BRDT*, *CEP120*, *BRCA1*	Regulate spermatogenesis and DNA repair	[31]
**Hu sheep**	*Lnc_001782*, *APOA4*, *APOA5*, oar-miR-411a-5p, *SMAD2*	*Lnc_001782* regulates *APOA4* and *APOA5* in lipid metabolism and liver function under heat stress, while *oar-miR-411a-5p* targets *SMAD2* to promote muscle growth and heat tolerance	[30]
**Egyptian Sheep**	*MYO5A*, *PRKG1*, *GSTCD*, *RTN1*, *ST3GAL3*, *PLCB1*, *STEAP3*, *KSR2*, *UNC13C*, *PEBP4*, *GPAT2*	*MYO5A*, *PRKG1*, *GSTCD*, *RTN1* regulate thermoregulation and oxidative stress; *ST3GAL3* enhances heat tolerance; *PLCB1*, *STEAP3*, *KSR2*, *UNC13C*, *PEBP4*, *GPAT2 * support metabolic and stress adaptation in hot climates	[29]
**Macheri sheep**	*HSF-2*, *HSF-5*	Regulate heat shock response and protein homeostasis	[27]
**Iranian sheep**	*MC1R*, *FOXN1*, *AZIN2*, *PPP1CC*, *CHMP1A*	Regulate coat color, cardiovascular function, and heat adaptation	[32]

**Table 6:** Overview of known genes under local adaptation for drought in sheep populations.

Pouplation	Genes	Function	References
**Kazakh sheep**	*TBXT*, *TG*, *HOXA1*	Regulate skeletal and developmental adaptations for drought-prone environments	[33]
**Egyptian fat-tail sheep**	*BMP7*, *MKNK1*, *PCK1*, *ACAA2*, *MAP3K2*, *PRDX1*, *TP53INP1*	These genes are crucial for water conservation, metabolic adaptation, and oxidative stress resistance, ensuring survival in dryland environments	[36]
**Sheep in the Taklimakan desert region**	*GPX3*, *GPX7*, *ANXA6*, *PTGS2*, *CPA3*, *CPVL*, *ECE1*, *CALM2*, *CACNA2D1*, *KCNJ5*, *COX2*, *AP1A*, *SLC4A4*, *CPA3*, *CPB1*	These genes collectively regulate water–salt metabolism, renal vasodilation, oxidative stress resistance, osmotic balance, and nutrient absorption, enhancing sheep adaptation to desert environments.	[34]
**Indigenous sheep in Xinjiang**	*SUCLG2*, *BMP2*, *TSHR*, *BANK1*	*SUCLG2* and *BMP2* support metabolic and skeletal adaptations to drought, while *TSHR* and *BANK1* enhance heat tolerance and feed efficiency.	[35]

## Abbreviations

The following abbreviations are used in this manuscript:

HIF-1α	Hypoxia-Inducible Factor 1-Alpha
EPAS1	Endothelial PAS Domain Protein 1
UCP1	Uncoupling Protein 1
SLC4A4	Solute Carrier Family 4 Member 4
GPX3	Glutathione Peroxidase 3
SOCS2	Suppressor of Cytokine Signaling 2
SOD1	Superoxide Dismutase 1
GPX4	Glutathione Peroxidase 4
HSP70	Heat Shock Protein 70
BMP2	Bone Morphogenetic Protein 2
BMP4	Bone Morphogenetic Protein 4
VEGFA	Vascular Endothelial Growth Factor A
EGLN1	Egl-9 Family Hypoxia-Inducible Factor 1
PPARGC1A	Peroxisome Proliferator-Activated Receptor Gamma Coactivator 1-Alpha
GDF9	Growth Differentiation Factor 9
BMPR1B	Bone Morphogenetic Protein Receptor Type 1B
FSHR	Follicle-Stimulating Hormone Receptor
MC1R	Melanocortin 1 Receptor
MITF	Microphthalmia-Associated Transcription Factor
LEF1	Lymphoid Enhancer-Binding Factor 1
PRDM16	PR/SET Domain 16
ATP2A1	Sarcoplasmic/Endoplasmic Reticulum Calcium ATPase 1
ADRB3	Beta-3 Adrenergic Receptor
PPARγ	Peroxisome Proliferator-Activated Receptor Gamma
AMPK	AMP-Activated Protein Kinase
JAK/STAT	Janus Kinase/Signal Transducers and Activators of Transcription
NF-κB	Nuclear Factor Kappa B
mTOR	Mechanistic Target of Rapamycin
AQP	Aquaporin
FOXO1	Forkhead Box O1
NOS3	Nitric Oxide Synthase 3
PARP2	Poly(ADP-Ribose) Polymerase 2
DNAH9	Dynein Axonemal Heavy Chain 9
SDK1	Sidekick Cell Adhesion Molecule 1
ARMC3	Armadillo Repeat Containing 3
PRDM16	PR/SET Domain 16
COL6A3	Collagen Type VI Alpha 3 Chain
COL25A1	Collagen Type XXV Alpha 1 Chain
HSPA1A	Heat Shock Protein Family A (Hsp70) Member 1A
HSPB1	Heat Shock Protein Family B (Small) Member 1
HSPD1	Heat Shock Protein Family D (Hsp60) Member 1
HSF4	Heat Shock Transcription Factor 4
PMEL	Premelanosome Protein
MLANA	Melan-A
DDB2	Damage-Specific DNA Binding Protein 2
SOCS6	Suppressor of Cytokine Signaling 6
GLIS1	GLIS Family Zinc Finger 1
AADACL3	Arylacetamide Deacetylase Like 3
GPR179	G Protein-Coupled Receptor 179
